# Nomogram for predicting 5-year metabolic dysfunction-associated steatotic liver disease risk: retrospective cohort study

**DOI:** 10.1530/EC-24-0186

**Published:** 2024-07-13

**Authors:** Lei Gao, Wenxia Cui, Dinghuang Mu, Shaoping Li, Nan Li, Weihong Zhou, Yun Hu

**Affiliations:** 1 Department of Geriatrics, Nanjing Drum Tower Hospital Clinical College of Nanjing Medical University, Nanjing, China; 2 Department of Geriatrics, Nanjing Drum Tower Hospital Clinical College of Nanjing University of Chinese Medicine, Nanjing, China; 3 Department of Health Management Center, Nanjing Drum Tower Hospital Clinical College of Nanjing Medical University, Nanjing, China

**Keywords:** 5-year risk, metabolic dysfunction-associated steatotic liver disease, nomogram, predictive model, retrospective cohort study

## Abstract

**Objective:**

To create a nomogram-based model to estimate the Chinese population's 5-year risk of metabolic dysfunction-associated steatotic liver disease (MASLD).

**Methods:**

We randomly divided 7582 participants into two groups in a 7:3 ratio: one group was assigned to work with the training set, which consisted of 5307 cases, and the other group was assigned to validate the model using 2275 cases. The least absolute shrinkage and selection operator model was employed to ascertain the variables with the highest correlation among all potential variables. A logistic model was constructed by incorporating these selected variables, which were subsequently visualized using a nomogram. The discriminatory ability, calibration, and clinical utility of the model were assessed using the receiver operating characteristic (ROC) curve, calibration curve, and decision curve analysis (DCA).

**Results:**

During the 5-year follow-up, 1034 (13.64%) total participants were newly diagnosed with MASLD. Using eight variables (gender, body mass index, waist, hemoglobin, alanine aminotransferase, uric acid, triglycerides, and high-density lipoprotein), we built a 5-year MASLD risk prediction model. The nomogram showed an area under the ROC of 0.795 (95% CI: 0.779–0.811) in the training set and 0.785 (95% CI: 0.760–0.810) in the validation set. The calibration curves revealed a 5-year period of agreement between the observed and predicted MASLD risks. DCA curves illustrated the practicality of this nomogram over threshold probability profiles ranging from 5% to 50%.

**Conclusion:**

We created and tested a nomogram to forecast the risk of MASLD prevalence over the next 5 years.

## Introduction

In 2023, international authorities adopted the nomenclature metabolic dysfunction-associated steatotic liver disease (MASLD), which was originally referred to as non-alcoholic fatty liver disease (NAFLD) (1). The estimated prevalence of MASLD in the world is greater than 30% (2). MASLD is a multi-systemic disease affecting not only the liver but also extra-hepatic organs (3). A study conducted on a nationwide cohort found that patients with MASLD have a higher likelihood of developing cardiovascular disease (4). Further, multiple studies have shown that MASLD is associated with an increased risk of chronic kidney disease (CKD) (5, 6).

Therefore, MASLD is a problem that needs to be taken extremely seriously. We need to identify patients at risk of progressing to MASLD in healthy populations in a timely manner and intervene in advance, thereby reducing the physical damage to people and the healthcare burden of the disease. Liver biopsy histopathology is the current diagnostic gold standard for MASLD; however, this invasive procedure has limited applicability due to its limited indications and contraindications (7). Imaging methods such as liver ultrasound or MRI can be used to identify patients with fatty liver but at a higher cost. There is a great need to construct models to predict MASLD.

Previous studies have developed equations to predict the development of NAFLD. In clinical practice, the fatty liver index (FLI) makes use of information that is easily accessible, and it exhibits a moderate level of predictive value (the area under the receiver operating characteristic (AUC): 0.84) (8). With an AUC of 0.812, the hepatic steatosis index (HSI) was also developed as an equation utilizing basic clinical signs (9). FLI and HSI have been demonstrated to be simple and efficient screening tools for NAFLD. In addition, there are a number of graphical models that may be used to forecast NAFLD, such as those developed using data from American and Japanese cohorts (10, 11). In China, a series of multiple visualization models based on data from elderly population, non-obese population, and pre-diabetic populations have been proposed (12, 13, 14).

Numerous machine learning-based models have also surfaced in recent years as a result of the use of machine learning techniques in the medical profession. All of these models showed good NAFLD identification ability, but the data they used were cross-sectional (15, 16, 17, 18). Deng *et al.* constructed a NAFLD prediction model based on longitudinal physical examination data using the long short-term memory algorithm (19). Huang *et al.* used six machine learning methods to construct a 5-year NAFLD prediction model and discovered that in both internal and external validation sets, the logistic regression model produced the greatest forecast performance (20). All of these models demonstrated good prediction of NAFLD incidence risk. However, given that NAFLD has been renamed as MASLD, these models have not been further validated in the MASLD population.

Therefore, our aim was to develop a nomogram for the 5-year MASLD risk using data obtained from Chinese health checkup records. We utilized receiver operating characteristic (ROC) curve, calibration curve, and decision curve analysis (DCA) to evaluate the model’s capacity to discriminate, calibrate, and provide clinical utility.

## Methods

### Study participants

A total of 12,312 participants underwent health checkups at the Nanjing Drum Tower Hospital Health Management Center in both 2018 and 2023. Data on smoking and drinking habits, as well as past medical history, were gathered from the participants. These are the criteria that were used to exclude candidates: participants who had been diagnosed with steatotic liver disease (SLD) at baseline; those who had viral hepatitis or other chronic liver diseases; those who had a history of heavy alcohol consumption (average daily alcohol consumption of more than 30 g for males and more than 20 g for females); and those who lacked the necessary information for this study.

Diagnostic criteria for MASLD were ultrasound diagnosis of steatosis of the liver and having at least one cardio-metabolic risk factor, while excluding a history of heavy alcohol consumption or other chronic liver diseases (1). Cardio-metabolic risk factors included: (1) blood pressure ≥ 130/85 mm Hg or specific antihypertensive drug treatment; (2) fasting plasma glucose (FPG) ≥ 5.6 mmol/L or 2-hour post-load glucose ≥ 7.8 mmol/L, hemoglobin A1c (HbA1C) ≥ 5.7%, or type 2 diabetes or treatment for type 2 diabetes; (3) body mass index (BMI) ≥ 23 kg/m² or waist > 94 cm (male) 80 cm (female); (4) plasma high-density lipoprotein (HDL) ≤ 1.0 mmol/L (male) and ≤1.3 mmol/L (female) or lipid-lowering treatment; (5) triglycerides (TG) ≥ 1.70 mmol/L or lipid-lowering treatment (1).

Hypertension was defined in our study as the following: (1) a previous definitive diagnosis of hypertension or the use of antihypertensive medication, or (2) an average of two blood pressure measurements were taken at the time of physical examination with a systolic blood pressure (SBP) of 140 mm Hg or higher or a diastolic blood pressure (DBP) of 90 mm Hg or higher. Diabetes was defined as the following: (1) a previous definitive diagnosis of diabetes or the use of hypoglycemic medication, or (2) FPG ≥ 7 mmol/L or HbA1C ≥ 6.5%.

**Figure 1:**
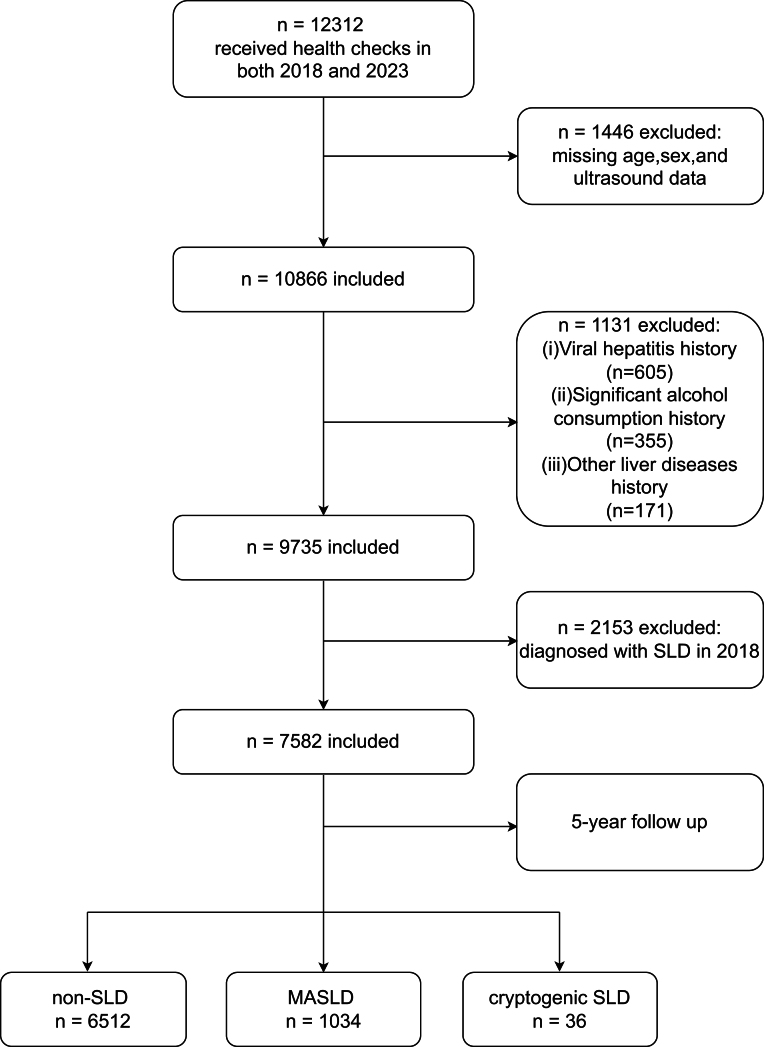
Flowchart of this study. SLD, steatotic liver disease; MASLD, metabolic dysfunction-associated steatotic liver disease.

A total of 7582 participants were enrolled in the study following the application of the exclusion criteria. Their average age was 39.62 ± 13.42 years, with 2870 males and 4712 females. After 5 years of follow-up, 1034 participants were diagnosed with MASLD and 36 with cryptogenic SLD. Figure 1 includes a detailed reference to the flowchart that depicts the enrolling process for research participants.

Written informed consent was obtained from all participants, and the study followed the ethical criteria of the Declaration of Helsinki. It was approved by the Ethics Committee of Nanjing Drum Tower Hospital (Ethics no. 2022-46-01).

### Variable characteristics

We used questionnaires, physical exams, and laboratory analysis as primary factors in this study. The questionnaires were used to collect information about the participants' ages, genders, medical backgrounds, and lifestyle behaviors, such as smoking and alcohol intake. Physical exams included measures of height, body weight, blood pressure, and waist circumference. Each person's BMI was determined by dividing their weight in kilograms by the square of their height in meters. Every participant refrained from eating overnight prior to the examination, and blood samples were collected from their peripheral veins in the early hours of the next day. Participants underwent standardized protocols at the same facility to assess their total blood count (Sysmex XN-1000; Sysmex Corp.) and blood biochemical index (Beckman AU5400; Beckman Coulter Corp.). Subsequently, we identified 19 variables with robust data quality and relevant physical measurements as potential variates.

### Ultrasound assessments for hepatic steatosis

Two skilled sonographers, who were not privy to the participants' medical records, used abdominal ultrasonography (Philips ultrasound equipment) to diagnose hepatic steatosis. Ultrasound criteria for identifying hepatic steatosis included the following: (1) parenchymal echoes in the liver are elevated (the intensity of the liver echo is greater than that in the spleen and kidneys); (2) the liver's far-field echoes are muted; (3) the intrahepatic venous conduits are not easily visible. The diagnosis of steatohepatitis can be made by the presence of item 1 of the above three items, plus any of items 2 or 3.

### Statistical analysis

The transparent reporting of a multivariable prediction model for individual prognosis or diagnosis recommendations is consistent with this research (21). To check if the data followed a normal distribution, the Kolmogorov–Smirnov test was run. Mean and s.d. were used to characterize normally distributed continuous variables, median and interquartile range were employed to characterize non-normally distributed continuous variables, and frequency and percentage were employed to characterize categorical variables. Depending on whether the continuous variables in the two groups had a normal distribution, we compared them using either an independent samples *t*-test or a rank sum test (Mann–Whitney *U* test). For categorical variables, the Chi-square test was employed. Multivariate logistic regression analysis was used to identify independent risk factors.

We performed sample size calculations using the three sample size criteria proposed by Riley *et al.* (22). The results can be found in Supplementary Table 1 (see section on supplementary materials given at the end of this article). Our sample size and the number of outcome events exceed the calculated sample size. Our study is therefore expected to provide reliable estimates. We used multiple imputations for the missing variables in this study.

Two sets, one for training and one for validation, were randomly assigned to participants in a 7:3 ratio. The least absolute shrinkage and selection operator (LASSO) regression method was used to select the optimal predictors for the study. The predictors selected by the LASSO regression model were then further statistically analyzed using multivariate logistic regression. Nomograms were used to visualize the results of the multiple logistic regression established above. The discriminatory power of the model for MASLD was assessed using the ROC curve to evaluate the AUC. The predicted probabilities were compared with the actual observed probabilities using a calibration curve. To evaluate the model's practicality in healthcare settings, DCA curves were employed.

We used R (version 4.3.1; https://www.R-project.org) to conduct our statistical analyses, and a *P* value of less than 0.05 was considered significant for two-sided statistical tests.

## Results

### Participant variables before and after follow-up

This study comprised 7582 participants who underwent a health checkup in 2018, excluding those with SLD or other liver diseases. Following a 5-year period, we collected health checkup data from the same study sample. New diagnoses of MASLD occurred in 1034 participants (13.64%) over the 5-year follow-up period. The comparison of variables between MASLD and non-MASLD groups categorized by outcome in 2018 and 2023 are presented in Table 1. In 2018, there was a higher likelihood of male participants (*P* < 0.001) and older participants (*P* < 0.001) who would experience MASLD 5 years later. Hypertension (*P* < 0.001) and diabetes mellitus (*P* < 0.001) were also found to have among the highest prevalence rates in this group. Furthermore, it should be mentioned that their BMI was significantly higher (*P* < 0.001), as was their waist circumference (*P* < 0.001), blood pressure (*P* < 0.001), and levels of liver enzymes (alanine aminotransferase (ALT), aspartate aminotransferase (AST), gamma-glutamyl transferase (GGT)), serum creatinine (Scr), uric acid (UA), total cholesterol (TC), triglycerides (TG), fasting plasma glucose (FPG), low-density lipoprotein (LDL), and hemoglobin (HB) were all elevated. Nevertheless, there was a significant decrease in their HDL levels (*P* < 0.001). Dissimilarities between the two categories persisted in 2023 as well.

**Table 1:** Comparison of variables between the MASLD and non-MASLD groups categorized by outcome in 2018 and 2023.

Variables	2018			2023		
	Non-MASLD (*n* = 6548)	MASLD (*n* = 1034)	*P*	Non-MASLD (*n* = 6548)	MASLD (*n* = 1034)	*P*
Gender (male%)	2217 (33.9%)	653 (63.2%)	<0.001	2217 (33.9%)	653 (63.2%)	<0.001
Age (years)	39.12 ± 13.38	42.68 ± 13.21	<0.001	44.12 ± 13.38	47.68 ± 13.21	<0.001
Hypertension (%)	579 (8.8%)	179 (17.3%)	<0.001	751 (11.5%)	265 (25.6%)	<0.001
Diabetes (%)	195 (3%)	51 (4.9%)	<0.001	249 (3.8%)	101 (9.8%)	<0.001
BMI (kg/m^2^)	22.17 ± 2.68	24.61 ± 2.59	<0.001	22.54 ± 2.71	25.93 ± 2.75	<0.001
Waist (cm)	75.13 ± 8.71	83.26 ± 8.24	<0.001	75.21 ± 9.36	86.62 ± 8.53	<0.001
SBP (mm Hg)	119.53 ± 15.85	125.60 ± 16.43	<0.001	123.23 ± 16.94	132.48 ± 17.10	<0.001
DBP (mm Hg)	74.44 ± 10.16	78.39 ± 10.93	<0.001	76.49 ± 10.44	82.34 ± 10.36	<0.001
HB (g/L)	141.50 ± 14.29	149.76 ± 14.33	<0.001	139.02 ± 14.86	149.02 ± 14.84	<0.001
ALT (IU/L)	15.80 (12.50, 21.20)	20.60 (16.10, 28.10)	<0.001	15.20 (11.50, 21.00)	24.00 (17.70, 34.10)	<0.001
AST (IU/L)	18.10 (15.70, 21.10)	19.80 (17.00, 23.00)	<0.001	17.90 (15.40, 21.10)	20.70 (17.60, 24.90)	<0.001
GGT (IU/L)	16.30 (12.90, 22.50)	22.20 (16.80, 33.10)	<0.001	18.30 (14.40, 25.40)	30.20 (22.00, 43.20)	<0.001
FPG (mmol/L)	4.94 ± 0.85	5.10 ± 0.84	<0.001	5.04 ± 0.88	5.44 ± 1.09	<0.001
Scr (µmol/L)	59.30 ± 13.86	65.89 ± 14.69	<0.001	61.28 ± 14.85	67.60 ± 14.95	<0.001
UA (µmol/L)	315.25 ± 78.77	367.89 ± 85.03	<0.001	315.38 ± 80.34	381.63 ± 84.80	<0.001
TG (mmol/L)	0.81 (0.60, 1.14)	1.21 (0.86, 1.64)	<0.001	0.88 (0.65, 1.27)	1.55 (1.15, 2.19)	<0.001
TC (mmol/L)	4.38 ± 0.81	4.53 ± 0.86	<0.001	4.89 ± 0.89	5.13 ± 0.98	<0.001
HDL (mmol/L)	1.46 ± 0.36	1.25 ± 0.31	<0.001	1.57 ± 0.39	1.27 ± 0.30	<0.001
LDL (mmol/L)	2.49 ± 0.68	2.71 ± 0.71	<0.001	2.83 ± 0.75	3.13 ± 0.82	<0.001

ALT, alanine aminotransferase; AST, aspartate aminotransferase; BMI, body mass index; DBP, diastolic blood pressure; FPG; fasting plasma glucose; GGT, gamma-glutamyl transferase; HB, hemoglobin; HDL, high-density lipoprotein; LDL, low-density lipoprotein; MASLD, metabolic dysfunction-associated steatotic liver disease; SBP, systolic blood pressure; Scr, serum creatinine; TG, triglycerides; TC, total cholesterol; UA, uric acid.

### Development of the predictive nomogram

We randomly divided the participants into a training set and a validation set in a 7:3 ratio. Table 2 shows a similar distribution of variables between these two groups.

**Table 2:** Comparison of variables between training set and validation set in 2018.

Variables	Training set (*n* = 5307)	Validation set (*n* = 2275)	*P*
Gender (male%)	1977 (37.3%)	893 (39.3%)	0.105
Age (years)	39.57 ± 13.40	39.70 ± 13.45	0.693
Hypertension (%)	521 (9.8%)	237 (10.4%)	0.449
Diabetes (%)	169 (3.2%)	77 (3.4%)	0.704
BMI (kg/m^2^)	22.50 ± 2.82	22.51 ± 2.74	0.857
Waist (cm)	76.21 ± 9.12	76.29 ± 9.01	0.714
SBP (mm Hg)	120.25 ± 16.05	120.61 ± 16.09	0.373
DBP (mm Hg)	75.03 ± 10.35	74.86 ± 10.38	0.521
HB (g/L)	142.50 ± 14.42	142.92 ± 14.94	0.248
ALT (IU/L)	16.40 (12.70, 22.20)	16.60 (12.80, 22.40)	0.730
AST (IU/L)	18.30 (15.80, 21.40)	18.40 (15.90, 21.60)	0.881
GGT (IU/L)	17.00 (13.20, 23.80)	16.80 (13.20, 23.65)	0.709
FPG (mmol/L)	4.96 ± 0.82	4.97 ± 0.91	0.777
Scr (µmol/L)	59.98 ± 14.13	60.73 ± 14.21	0.034
UA (µmol/L)	322.00 ± 81.97	323.44 ± 80.98	0.481
TG (mmol/L)	0.86 (0.62, 1.24)	0.85 (0.62, 1.22)	0.656
TC (mmol/L)	4.40 ± 0.83	4.39 ± 0.79	0.628
HDL (mmol/L)	1.43 ± 0.37	1.43 ± 0.35	0.929
LDL (mmol/L)	2.52 ± 0.70	2.52 ± 0.67	0.903

ALT, alanine aminotransferase; AST, aspartate aminotransferase; BMI, body mass index; DBP, diastolic blood pressure; FPG, fasting plasma glucose; GGT, gamma-glutamyl transferase; HB, hemoglobin; HDL, high-density lipoprotein; LDL, low-density lipoprotein; MASLD, metabolic dysfunction-associated steatotic liver disease; SBP, systolic blood pressure; Scr, serum creatinine; TG, triglycerides; TC, total cholesterol; UA, uric acid.

**Figure 2:**
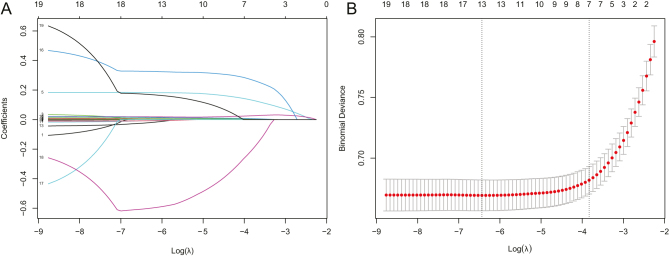
Selection of predictor variables via least absolute shrinkage and selection operator (LASSO) regression. (A) Regression coefficients plotted against Log(*λ*). (B) Mean square error in LASSO regression depicted as a function of Log(*λ*).

The initial univariate analysis of rows within the training set indicated a total of 19 variables. These variables were gender, age, BMI, waist, history of hypertension and diabetes, SBP and DBP, HB, ALT, AST, GGT, Scr, FPG, UA, TG, TC, LDL, and HDL. Subsequently, we conducted LASSO regression analyses using a regularization parameter value (*λ*) of 0.0149. Figure 2 provides a visual representation of the correlation between the regression coefficients and Log *λ*. A total of eight variables were culled from the original set of 19 by this analysis. Gender, BMI, waist, ALT, HB, UA, TG, and HDL were used as characteristic variables to construct a 5-year MASLD risk prediction model. The logistic regression model's variables are shown in Table 3, along with their regression coefficients, odds ratios, and 95% CI.

**Table 3:** Multivariate logistic regression analysis for the prediction of MASLD in the training set.

Variables	*B*	OR (95% CI)	*P*
Gender (male)	−0.30	0.74 (0.54–1.01)	0.059
BMI (kg/m^2^)	0.19	1.21 (1.14–1.28)	<0.001
Waist (cm)	0.02	1.02 (1.00–1.04)	0.034
HB (g/L)	0.02	1.02 (1.01–1.02)	<0.001
ALT (IU/L)	0.01	1.01 (1.01–1.02)	<0.001
UA (µmol/L)	0.002	1.00 (1.00–1.00)	0.002
TG (mmol/L)	0.42	1.52 (1.34–1.72)	<0.001
HDL (mmol/L)	−0.54	0.58 (0.43–0.78)	<0.001

ALT, alanine aminotransferase; BMI, body mass index; HB, hemoglobin; HDL, high-density lipoprotein; MASLD, metabolic dysfunction-associated steatotic liver disease; OR, odds ratio; TG, triglycerides; UA, uric acid.

**Figure 3:**
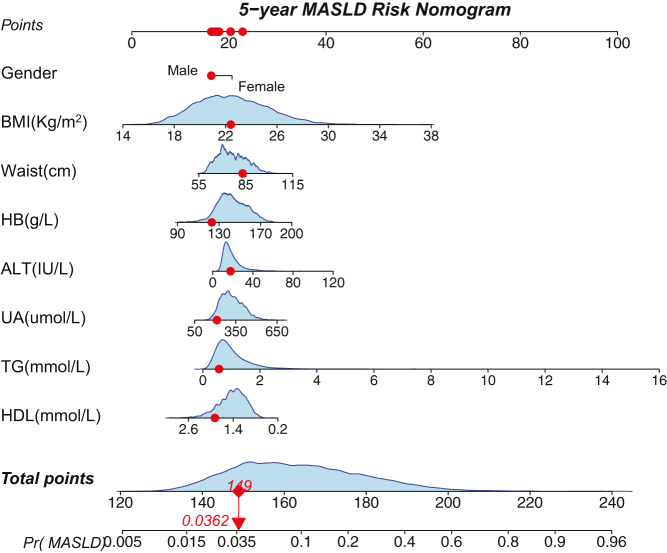
Nomogram for predicting 5-year metabolic dysfunction-associated steatotic liver disease (MASLD). BMI, body mass index; HB, hemoglobin; ALT, alanine aminotransferase; UA, uric acid; TG, triglycerides; HDL, high-density lipoprotein.

A nomogram was used to graphically display the logistic regression model. Figure 3 illustrates the nomogram. For example, when a healthy male has a BMI of 22.5 kg/m^2^, waist circumference of 85 cm, HB of 120 g/L, ALT of 20 IU/L, UA of 200 μmol/L, TG of 0.5 mmol/L, and HDL of 2.0 mmol/L, after 5 years, his probability of having MASLD is 3.62%, according to our calculations.

### Internal validation of predictive model

**Figure 4:**
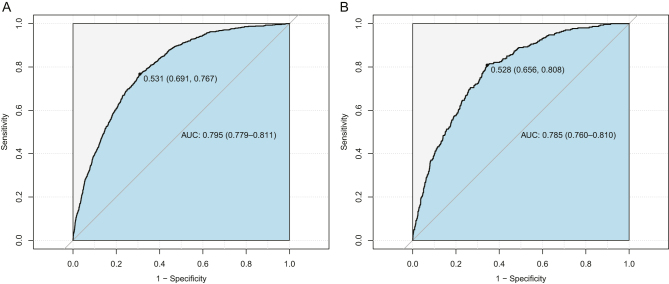
Receiver operating characteristic (ROC) curves. (A) From the training set and (B) from the validation set. AUC, area under the ROC.

**Figure 5:**
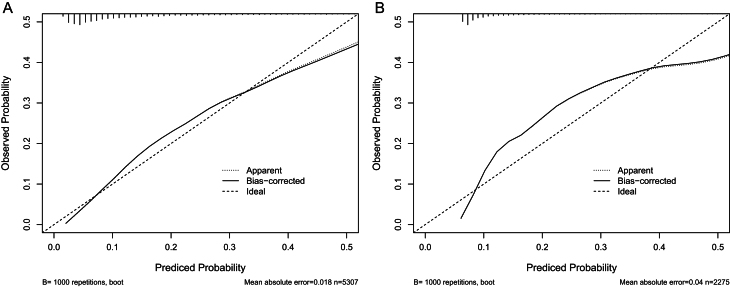
Calibration curves of the nomogram. (A) From the training set and (B) from the validation set.

**Figure 6:**
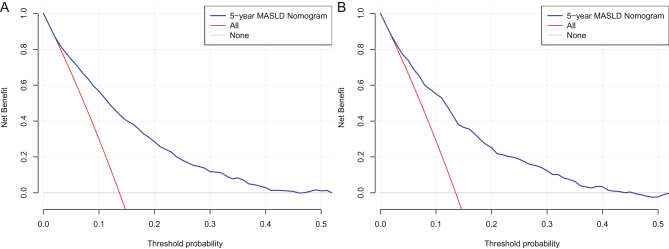
Decision curves of the nomogram. (A) From the training set and (B) from the validation set. MASLD, metabolic dysfunction-associated steatotic liver disease.

Figure 4 illustrates the ROC curves for the nomogram. The AUC of the training set is 0.795 (95% CI: 0.779–0.811). The AUC of the validation set is 0.785 (95% CI: 0.760–0.810). This study proves with its findings that the nomogram has a strong discriminatory performance in predicting the development of MASLD over a period of 5 years. In order to evaluate the degree of congruence between the predicted values derived from the nomogram and the actual incidences of MASLD, calibration curves were utilized. These curves are displayed in Fig. 5. Furthermore, the results of the DCA, which are depicted in Fig. 6, demonstrated that the nomogram's clinical value was superior to the scenarios of ‘none developed MASLD’ or ‘all developed MASLD’. The threshold probability for the predictive model is 5–50%.

## Discussion

We followed 7582 participants in health checkups for 5 years. During the follow-up, 1034 (13.64%) participants were newly diagnosed with MASLD. Using eight variables (gender, BMI, waist, ALT, HB, UA, TG, and HDL) filtered by LASSO regression, we built a 5-year MASLD risk prediction model. The nomogram showed an AUC of 0.795 (95% CI: 0.779–0.811) in the training set and 0.785 (95% CI: 0.760–0.810) in the validation set. The calibration curves revealed a 5-year period of agreement between the observed and predicted MASLD risks. DCA curves demonstrated the clinical utility of this nomogram over threshold probability profiles ranging from 5% to 50%.

Our study identified eight variables for predicting 5-year MASLD risk as gender, BMI, waist, ALT, HB, UA, TG, and HDL. A growing number of people use the FLI (8), which is based on cross-sectional data and includes variables like waist, BMI, TG, and GGT. BMI, waist, and TG are included as predictors in our longitudinal MASLD prediction model, confirming the strong association between MASLD and these variables. This further suggests that the new name MASLD reflects the pathogenesis better than NAFLD. In our study, both LDL and HDL were correlated with the development of MASLD in univariate analysis. LDL was excluded from further multivariate regression analysis. Nonetheless, HDL levels were still negatively correlated with MASLD risk. A recent study using liquid chromatography–mass spectrometry found that the HDL content of patients with MASLD was decreased, and the lipid composition of HDL differed from that of control subjects (23).

A variety of liver disorders, including fibrosis, cirrhosis, and hepatocellular carcinoma, can progress in MASLD. The severity of these conditions can vary from mild steatosis to life-threatening steatohepatitis (24). When it comes to liver disease in Americans waiting for a transplant, nonalcoholic steatohepatitis ranks second (25). Not only that, but MASLD increases the risk of extrahepatic disease. Mounting evidence has linked MASLD to an elevated risk of cardiovascular disease, including both fatal and non-fatal complications (26, 27). Additionally, the level of seriousness of MASLD and a higher prevalence of cardiovascular problems are correlated (28). Several studies conducted in both hospitals and populations have demonstrated that the incidence of CKD is significantly higher in patients who have MASLD, and that the association between MASLD and CKD is significant even after accounting for hypertension, type 2 diabetes, and other risk factors for CKD (6, 29). There is also an increased likelihood of developing colorectal adenomas and osteoporosis when MASLD is present (30, 31). The amount of money spent annually on the treatment of MASLD in the United States is over one hundred billion dollars, whereas in four European countries (the United Kingdom, France, Germany, and Italy) it is over 35 billion Euros (32).

As the global obesity problem continues to grow, the prevalence of MASLD is gradually increasing (7). Considering the high prevalence and potential harm of MASLD, it is especially crucial to identify high-risk individuals at an early stage in healthy populations. Although several models for predicting NAFLD have been proposed in the past, most of them have not been revalidated in the MASLD population, and most of these models are constructed based on cross-sectional data. Our study, based on a longitudinal follow-up cohort of the population, developed a predictive model that can be applied when individuals have not yet been diagnosed with MASLD. At the same time, our model variables are relatively simple and applicable to mass screening. By presenting the model results through nomogram-based visualizations, our study enables participants to more easily understand their own risk of developing MASLD, thus facilitating individual self-awareness and lifestyle changes. All of this contributes to reducing the risk of developing MASLD and further reducing the harm it causes.

Nonetheless, several limitations must be considered: (1) we used ultrasound results of the liver to diagnose MASLD, rather than the results of a liver biopsy, which was biased because the gold standard method was not used. (2) Logistic regression analysis was utilized in lieu of COX regression analysis due to a lack of follow-up duration information. (3) The nomogram was developed using data from a single center. (4) The results of this study had only been validated with an internal validation set. (5) Our study lacked factors regarding demographic characteristics, dietary preferences, exercise habits, and medication history, which can influence the onset of MASLD. Due to the lack of these factors, this may limit the predictive ability of our prediction model.

## Conclusion

A predictive nomogram was constructed incorporating eight risk predictors (gender, BMI, waist, ALT, HB, UA, TG, and HDL) for anticipating MASLD occurrence over a 5-year period. This nomogram serves as a straightforward and economical tool to identify high-risk individuals for MASLD, enabling timely interventions aimed at mitigating the onset of the disease.

## Supplementary Materials

Supplemental Table 1. Minimum Sample Size Required to Develop a Multivariable Prediction Model for a Binary Outcome using pmsampsize package in RStudio

## Declaration of interest

The authors maintain that the study is devoid of any conflicts of interest that could potentially compromise its objectivity.

## Funding

The funding for this research came from the Nanjing Health Science and Technology Developmenthttp://dx.doi.org/10.13039/100006180 Major Project no. ZDX21001.

## Author contribution statement

YH and WZ were responsible for the preliminary design and development of the study. L G gathered information, conducted additional data and statistical analysis, and penned the article. The article was conceptualized and substantially revised by WC. Both NL and DM contributed to the article revision and data acquisition processes. All contributors approved and co-authored the manuscript.
