# Supplementary material for: Nomogram for predicting 5-year metabolic dysfunction-associated steatotic liver disease risk: retrospective cohort study
Source: Endocr Connect. 2024 Jul 13;13(8):e240186. doi: 10.1530/EC-24-0186 (PMC11301545; doi:10.1530/EC-24-0186)
Supplement: Supplemental Table 1. Minimum Sample Size Required to Develop a Multivariable Prediction Model for a Binary Outcome using pmsampsize package in RStudio [file supplementary_table_1.pdf]

Supplemental Table 1. Minimum Sample Size Required to Develop a Multivariable Prediction Model for a Binary Outcome using *pmsampsize* package in RStudio

	**Minimum Sample Size Required**	**Shrinkage**	**Cox-Snell R^2**
**Candidate Predictors (n=19 parameters)**			
Criteria 1[a]	607	0.90	0.2421
Criteria 2[b]	447	0.87	0.2421
Criteria 3[c]	350	0.90	0.2421
**Final Model Predictors (n=4 parameters)**			
Criteria 1[a]	256	0.90	0.2421
Criteria 2[b]	189	0.87	0.2421
Criteria 3[c]	350	0.90	0.2421

[a]Criteria 1: small overfitting defined by an expected shrinkage of predictor effects by 10% or less
[b]Criteria 2: small absolute difference of 0.05 in the model's apparent and adjusted R-squared value
[c]Criteria 3: precise estimation (within +/- 0.05) of the average outcome risk in the population
[1]

[1] With 19 candidate predictor parameters,we estimated we would need a minimum sample size of 607 with 213 events to meet all three sample size criteria proposed by Riley et. Al(22).

With 8 predictor parameters, as in our final model, a slightly smaller sample size is required (N=350).
Our sample size is sufficient to fulfill the first two criteria proposed by Riley et. al criteria.
